# Interactions between key genes and pathways in prostate cancer progression and therapy resistance

**DOI:** 10.3389/fonc.2025.1467540

**Published:** 2025-01-23

**Authors:** Fan Wu, Hengsen Zhang, Miaomiao Hao

**Affiliations:** ^1^ Department of Pathology, Shanghai Ninth People’s Hospital, School of Medicine, Shanghai Jiao Tong University, Shanghai, China; ^2^ Department of Neurosurgery, Affiliated Hospital of Jiangnan University, Wuxi, China

**Keywords:** signaling pathway, therapeutic targets, CRPC, prostate cancer, gene mutation

## Abstract

Prostate cancer is one of the most prevalent malignant tumors in men, particularly in regions with a high Human Development Index. While the long-term survival rate for localized prostate cancer is relatively high, the mortality rate remains significantly elevated once the disease progresses to advanced stages, even with various intensive treatment modalities. The primary obstacle to curing advanced prostate cancer is the absence of comprehensive treatment strategies that effectively target the highly heterogeneous tumors at both genetic and molecular levels. Prostate cancer development is a complex, multigenic, and multistep process that involves numerous gene mutations, alteration in gene expression, and changes in signaling pathways. Key genetic and pathway alterations include the amplification and/or mutation of the androgen receptor, the loss of Rb, PTEN, and p53, the activation of the WNT signaling pathway, and the amplification of the MYC oncogene. This review summarizes the mechanisms by which these genes influence the progression of prostate cancer and highlights the interactions between multiple genes and their relationship with prostate cancer. Additionally, we reviewed the current state of treatments targeting these genes and signaling pathways, providing a comprehensive overview of therapeutic approaches in the context of prostate cancer.

## 1 Introduction

Prostate cancer is currently the most common malignancy among men in the United States, with an incidence of 29% (1). In 2024, it is the most common cause of male cancer death after lung and bronchial cancer (1). Globally, prostate cancer mortality is slightly lower than that of lung cancer in the male population (2). Therefore, prostate cancer ranks high in both incidence and mortality rates. Prostate cancer is influenced by various risk factors, including age, family history, obesity, and unhealthy dietary habits (3). Age is a primary risk factor for prostate cancer. The incidence is rare in men under 50 years old (1 in 350), but it increases sharply to 1 in 52 by age 59, and by age 65, the rate rises to more than 1 in 2. Men with a family history of the disease have more than double the risk of developing prostate cancer compared to those without such a history (4–6). Additionally, race plays a role in prostate cancer risk. Research from 2010 found, compared to White male patients, Black male patients exhibit a more rapid progression of prostate cancer and may develop invasive prostate cancer at an earlier stage (7, 8).

Early-stage prostate cancer often lacks noticeable symptoms, making it difficult to detect and delaying timely and effective treatment. Currently, the screening and diagnosis of prostate cancer mainly include serum Prostate-specific Antigen (PSA), Magnetic Resonance Imaging fusion ultrasound-guided prostate biopsy (MRI-TRUS), and digital rectal examination. Despite the availability of these methods, PSA remains the most widely used screening tool for early diagnosis of prostate cancer worldwide. Although PSA is highly sensitive for early detection, it lacks specificity of the properties of prostate tissue. This means it cannot differentiate between high-risk and low-risk tumors and may also be elevated in cases of enlarged prostate, aging, prostatitis, certain urological diseases, and specific drug treatments. Consequently, PSA screening may lead to overtreatment of prostate cancer (9).

In recent years, alongside PSA, other tumor markers such as p53, MDM2 and Ki67 have been used to monitor the progression and treatment of prostate cancer. Additionally, the application of next generation sequencing (NGS) technology in cancer diagnosis and treatment has deepened researchers’ understanding of prostate cancer and its molecular biology. Drug therapies targeting prostate cancer-related genes are also under investigation and some of them have been used in clinical treatment, but none of the therapeutic effects are very satisfactory, and the treatment of advanced prostate cancer is still an urgent problem to be solved. This article primarily reviews the treatment, drug resistance, and prognosis of genes related to prostate cancer. The interactions between related genes are further summarized and it is suggested that combination therapy targeting such multiple genes may be more effective in the treatment of advanced prostate cancer.

## 2 Androgen receptor

### 2.1 Role of AR in prostate cancer

Androgen receptor (AR), a nuclear transcription factor in the steroid hormone receptor family, is central to prostate cancer pathogenesis. When testosterone or 5-alpha-dihydrotestosterone (DHT) binds to AR, the receptor dimerizes and translocations to the nucleus, where it binds to the androgen response element (ARE) (10). This interplay participates in the transcriptional activity of genes that prevent apoptosis and induce cell proliferation. AR supports proper development in normal prostate, whereas elevated AR expression drives disease progression in prostate cancer (11).

### 2.2 Mechanisms of resistance to ADT

**Figure 1:**
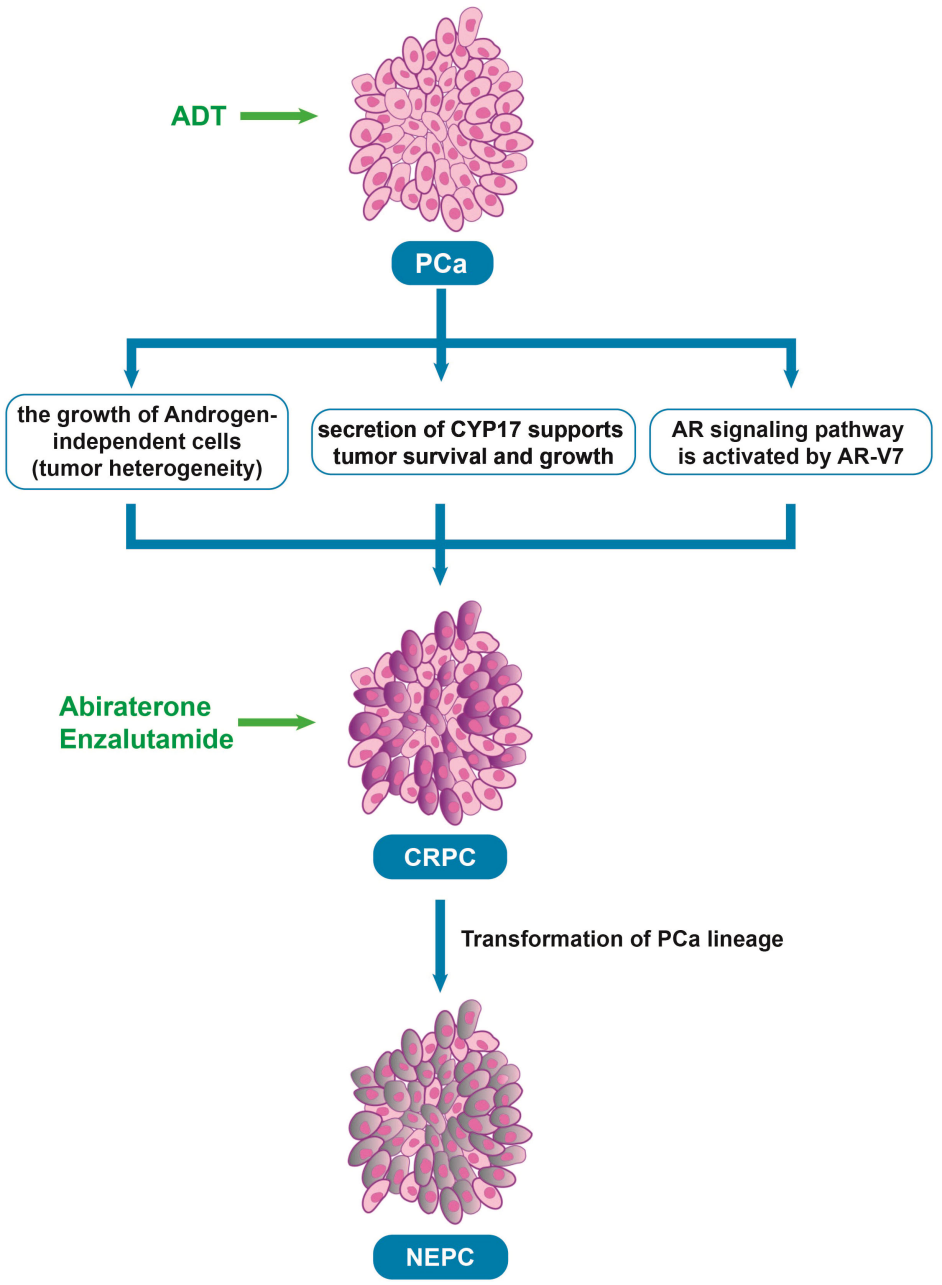
The process underlying the development of CRPC after ADT. PCa, Prostate cancer; ADT, Androgen deprivation therapy; CRPC, Castrate resistant prostate cancer; AR, Androgen receptor; NEPC, neuroendocrine prostate cancer, Abiraterone and Enzalutamide: The androgen biosynthesis inhibitor and the novel AR inhibitor.

Androgen deprivation therapy (ADT) is a treatment designed to reduce or block the production of androgens (male hormones, such as testosterone) that fuel the growth of prostate cancer. ADT is initially effective in treating prostate cancer (12). As the disease progresses, most patients eventually develop castrate-resistant prostate cancer (CRPC) and metastases after ADT (**Figure 1**). There are two main mechanisms behind this resistance. First, although early-stage prostate cancer is primarily driven by androgen-dependent cancer cells, the disease is heterogeneous, not only composed of androgen-dependent cells. Castration resistance occurs due to the growth of androgen-independent cells, which arises from genetic alterations in the AR (13). Second, apart from the androgens produced by the adrenal glands and testis that stimulate AR, intra-tumoral secretion of enzymes involved in testosterone synthesis, such as cytochrome P450 17-alpha hydroxysteroid dehydrogenase (CYP17), also supports tumor survival and growth (14). Moreover, a new mechanism about the resistance has been found in recent years. AR splice variants are more common in CRPC, and they are characterized primarily by the loss of ligand domains, which retain the ability to bind to DNA in the absence of androgens (15). There are many variants of AR spliceosome, among which AR-V7 is one of the most studied variants. AR-V7 can complete nuclear transfer in the absence of androgen binding and recruit cofactors to complete transcriptional activation of downstream genes, followed by aberrant activation of the AR signaling pathway (16). Interestingly, AR-V7 also predicted treatment response to AR-targeting drugs, and AR-V7-positive patients who received enzalutamide and abiraterone had shorter progression-free survival and shorter overall survival than AR-V7-negative patients (17). This also provides strong evidence for AR-V7 as a biomarker for prostate cancer.

### 2.3 Emerging therapies and challenges

To target CRPC, new drugs that inhibit androgen-producing enzymes or block AR have been developed in recent years, such as second-generation nonsteroidal AR antagonists (enzalutamide, apalutamide, and darolutamide) and the androgen biosynthesis inhibitor abiraterone (18). In a phase 3 trial of enzalutamide, which randomized 1,125 male patients with metastatic castration sensitive prostate cancer (mCSPC) into groups of ADT in combination with either enzalutamide (N = 563) or a standard nonsteroidal antiandrogen agent (bicalutamide, flutamide, or nilutamide; N = 562) until progression or unacceptable toxicity. the enzalutamide arm had fewer deaths than the standard-care group (102 *vs* 143; HR 0.67; 95% CI 0.52-0.86; *P* = 0.002) and 3-year overall survival (OS) estimated at 80% (based on 94 events) *vs* 72% (based on 130 events), respectively (19). In another clinical trial, 297 patients with high-risk metastatic hormone-sensitive prostate cancer (mHSPC) treated with abiraterone, 127 with enzalutamide, and 142 with apalutamide were compared. There were no differences in time to CRPC (p = 0.13), OS (p = 0.7), and cancer-specific survival (CSS) (p = 0.5) among the three ARPIs, but abiraterone was significantly better in 99% PSA decline achievement compared to apalutamide (72% vs. 57%, p = 0.003) (20). However, over time, most patients still develop resistance to these treatments (**Table 1**) (21). Some studies have found that after treatment with anti-androgen drugs, prostate cancer cells undergo a lineage shift, which refers to the conversion of cells from luminal and basal cells to neuroendocrine-type cells caused by adaptation to the environment (22–24). Thus, the prostate cancer cells can evade drug-targeted therapy, causing treatment-resistant neuroendocrine prostate cancer.

**Table 1:** Genes associated with prostate cancer progression.

Gene	Function	Interaction between genes
AR	Regulation of AR signaling pathway	Rb, p53, MYC, WNT
Rb	Regulation of cell cycle	AR, p53, PTEN
PTEN	Regulation of PI3K/AKT signaling pathway	Rb, p53, MYC
WNT	Regulation of WNT/β-catenin signaling pathway	AR
p53	Regulation of cell cycle	Rb, PTEN, AR
MYC	Regulation of gene expression and key cellular processes	AR, PTEN

## 3 Retinoblastoma

### 3.1 Mechanisms of cell cycle regulation by retinoblastoma

Retinoblastoma is a malignant tumor, and Retinoblastoma (Rb) is a tumor suppressor gene identified in this tumor (25, 26). The Rb gene is located on chromosome 13q14.2 and was the first human tumor suppressor gene to be cloned (25). The Rb protein family includes Rb, p107 and p130, collectively referred to as “pocket proteins”, which are involved in cell cycle regulation (27). The cell cycle is the series of events in which cellular components are doubled, and then accurately segregated into daughter cells. In eukaryotes, the cell cycle consists of four phases, S-phase, in which DNA replication occurs, M-phase, in which mitosis occurs, and two interphases, G1 and G2, between S-phase and M-phase, which are the times when the cell acquires mass, integrates growth signals, organizes the replication of the genome, and prepares the chromosomes for segregation (28). In its low phosphorylation state, Rb can inhibit the transcriptional activity of E2F by binding to its downstream transcription factors (E2F), thereby suppressing the expression of genes involved in the cell cycle and arresting the cell cycle in the G1 phase (29). However, in late G1, Rb transitions from a low phosphorylation state to a high phosphorylated, inactive state, releasing E2F and allowing cells to enter the S-phase, thereby promoting cell proliferation (29). The cyclin-cyclin dependent kinase (CDK) complex promotes cell cycle progression by phosphorylating members of the Rb family during G1. Cyclin D expression leads to CDK4 (and CDK6)- dependent phosphorylation of Rb, reducing its binding to E2Fs and promoting early cell cycle gene expression (30). CDK inhibitors (such as p16 and p21) can prevent CDK from phosphorylating Rb by inhibiting the activity of CDK4 and CDK6, thereby promoting Rb function (31).

### 3.2 Role of retinoblastoma in prostate cancer

The inactivation of Rb is closely related to all stages of prostate cancer formation (32). Rb-mediated loss of cell cycle control only leads to the occurrence of prostatic proliferative diseases and is not sufficient to cause malignant tumors (33). It has been shown that Rb deletion can promote angiogenesis, metastasis and neuroendocrine differentiation (NED), a process by which epithelial tumor cells acquire features of neuroendocrine cells, resulting in a more aggressive phenotype in human prostate cancer cells (34). In addition, Rb can promote epithelial-mesenchymal transition (EMT) and tumor cell invasion by regulating downstream target genes (35). Recently, Jin, X., et al. reported that the Rb-NF-κB axis can be used to overcome cancer immune escape induced by conventional or targeted therapies (36). Thus, while the absence of Rb does not cause the occurrence of prostate cancer, it can lead to the proliferation of prostate cells and plays an essential role in the metastasis, EMT and NED of prostate cancer.

**Figure 2:**
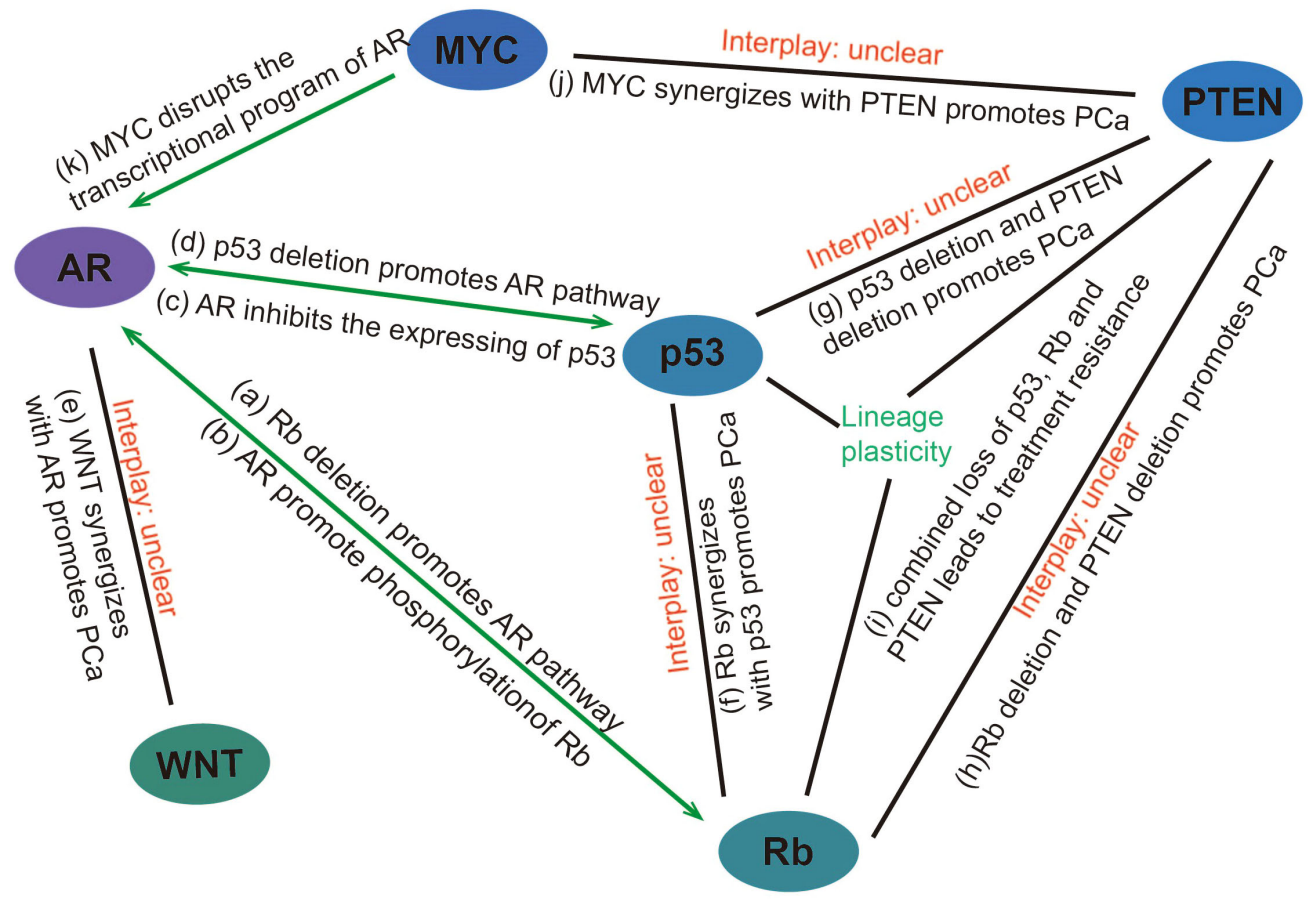
Interaction between genes/pathways in prostate cancer **(A)** Rb deletion promotes the expression of genes downstream of the AR pathway through transcriptional activation of E2F and facilitating promoter recruitment of the AR. **(B)** AR induces signals that promote CDK activity and promotes phosphorylation of Rb to inactivate it. **(C)** AR can promote prostate cancer progression by reducing p53 expression through G3BP3, which promotes the nuclear translocation of P53. **(D)** p53 overexpression inhibits androgen-induced transactivation of NKX3.1 by repressing the promoter of the AR gene and blocking AR-DNA binding activity. Conversely, p53 deletion promotes prostate cancer progression by facilitating the AR signaling pathway. **(E)** WNT can synergize with AR to promote the development of aggressive prostate cancer. **(F)** p53 and Rb deletion can mediate lineage plasticity, thereby enabling prostate cancer to evade targeted therapies and progress to CRPC. **(G)** The deletion of PTEN and p53 can lead to changes in the lineage of prostate cancer, resulting in the formation of CRPC. **(H, I)** Rb loss facilitates lineage plasticity and metastasis of prostate adenocarcinoma initiated by PTEN mutation. The additional loss of p53 causes resistance to antiandrogen therapy. **(J)** MYC overexpression or targeted PTEN loss can each produce early prostate adenocarcinomas but are not sufficient to induce genetic instability or metastases with high penetrance. However, MYC activation and PTEN deletion induced genomic instability and aggressive prostate cancer. **(K)** MYC promotes the development of mCRPC by disrupting the transcriptional program of AR. PCa, Prostate cancer.

In addition to promoting the development of prostate cancer through the aforementioned mechanisms, Rb loss also participates in the AR signaling pathway. Androgens are known prostatic epithelial cell growth factors (37) and play an important role in prostate cancer development. Androgens can activate Rb by regulating CDK4/cyclin D1 and CDK2 complexes, thereby initiating the cell cycle (38). After androgen castration treatment, the level of cyclin D protein is reduced, maintaining low Rb phosphorylation, causing cell cycle arrest, and inhibiting tumor development (39). Sharma, A., et al. have found that CRPC that develops after castration-resistant treatment shows decreased Rb expression and increased AR expression (40). Subsequently, Gupta, S. et al. also have found that AR overexpression in CRPC was associated with Rb inactivation (41). we believe that there are several mechanisms for this phenomenon: 1) deletion of Rb activates E2F, which acts downstream of it to increase AR expression (42, 43); 2) Rb loss increases AR recruitment to homologous promoters, resulting in increased AR target gene expression (44); 3) AR induces signals that promote CDK activity and promotes phosphorylation of Rb to inactivate it (**Figure 2**) (45).

### 3.3 Emerging therapies and challenges

Given the above mechanism of cell cycle regulation by Retinoblastoma in prostate cancer, inhibition of Rb phosphorylation can be used as a therapeutic strategy for prostate cancer. By binding CDK inhibitors to CDK4 and CDK6, Rb phosphorylation is inhibited to prevent the G1-S phase transition and induce cell cycle arrest. At present, there is evidence that highly selective small molecule inhibitors of CDK4 and CDK6, Palbociclib, Ribociclib and Abemaciclib, are effective in the treatment of breast cancer (46), but the therapeutic effect of prostate cancer is not clear. In breast cancer, data from the latest MONARCH-3 study showed that at a median follow-up time of 8.1 years, treatment with Abemaciclib in combination with an nonsteroidal aromatase inhibitor (NSAI) numerically prolonged Overall Survival (OS) compared to NSAI therapy alone in patients with HR+, HER2- advanced breast cancer, however, unfortunately, the difference did not reach statistical significance (P=0.0664) (47). Ribociclib is the only CDK4/6 inhibitor that has achieved positive OS results in all three phase III studies, with stable and consistent OS benefit, whether targeting premenopausal or postmenopausal populations, as a first- or second-line treatment, or in combination with an aromatase inhibitor (AI) or fulvestrant. This is based on several unique mechanisms of action. Firstly, Ribociclib can induce tumors cell senescence to achieve a long-term response (48); secondly, Ribociclib significantly affects peripheral innate and adaptive immune responses, and achieves long-term efficacy through immune activation (49). These are all characteristics that Abemaciclib does not possess. Likely due to the above reasons, the most recent phase 3 study of Abemaciclib with abiraterone in patients with metastatic CRPC (mCRPC) did not show a significant increase in radiographic progression-free survival (rPFS) for the addition of Abemaciclib to abiraterone, the medians rPFS were 21.96 months for the Abemaciclib plus abiraterone group vs 20.28 months for the placebo (PBO) plus abiraterone group (50).

## 4 PTEN

### 4.1 Mechanisms of PTEN in cell proliferation and apoptosis

Phosphatase and tensin homolog gene (PTEN) is a tumor suppressor gene with phosphatase activity, which is located in chromosome 10q23 and spans 200kb in full length (51). PTEN is involved in tumor progress by inhibiting the phosphatidylinositol 3-kinase (PI3K)/serine-threonine kinase (AKT)/mammalian target of the rapamycin (mTOR) pathway and its reduction or loss of expression caused by methylation, mutation or deletion is closely related to the occurrence and development of various tumors (52). The PI3K/AKT/mTOR pathway is crucial for cell signal transduction. PTEN enables dephosphorylate phosphatidylinositol (3,4,5)-trisphosphate (PIP3) of PI3K/AKT/mTOR pathway to generate Phosphatidylinositol (4,5)-bisphosphate (PIP2). When PTEN is lost, PIP2 is phosphorylated to PIP3 by PI3K, activating a series of kinases in the signaling pathway, including AKT. AKT affects cell apoptosis through serine phosphorylation of Bcl-2-associated death promoter (BAD) and Caspase-9, and influences cell proliferation, differentiation, and survival through regulation of transcription, translation, and cell cycle (53).

### 4.2 Role of PTEN in prostate cancer

The loss of PTEN promotes overactivation of the PI3K/AKT/mTOR signaling pathway leading to cell transformation and tumorigenesis (54). In a study on the PTEN deleted mouse model of prostate cancer, a blockade of mTOR inhibited prostate tumorigenesis in epithelial cells (55). Numerous studies have found that the occurrence of prostate cancer and its hormone-independent transformation course are related to the loss of PTEN gene expression (56, 57). Studies have shown that PTEN is absent in 15% to 20% of primary prostate cancers, and the frequency of PTEN deletion is higher in CRPC and mCRPC tissues, reaching 40% to 60% (58). PTEN deletion is positively correlated with Gleason score, pathological grade, clinical stage and metastasis of prostate cancer (59). The above evidence suggests that PTEN loss is closely associated with prostate cancer progression and tumorigenesis.

### 4.3 Emerging therapies and challenges

Several inhibitors (rapamycin analogs) targeting the PI3K/AKT/mTOR pathway have been investigated to counteract the mechanism by which PTEN deletion promotes prostate cancer progression (60), but their antitumor effects have been disappointing. Although rapamycin inhibited PI3K/AKT/mTOR pathway, long-term treatment caused resistance and was not suitable for monotherapy. Wang Y. et al. have found that combination of rapamycin and bicalutamide (anti-androgenic drug) improved anti-prostate cancer effect due to the suppression of mTOR stimulated AR transcriptional activity (61). A clinical trial for mCRPC demonstrated that the combination of the PI3K inhibitor samotolisib with enzalutamide, which causes an improved PFS in mCRPC patients progressing on abiraterone, median Prostate Cancer Clinical Trials Working Group criteria (PCWG2)-PFS and rPFS was significantly longer in the samotolisib/enzalutamide versus placebo/enzalutamide arm (3.8 vs. 2.8 months; P = 0.003 and 10.2 vs. 5.5 months; P = 0.03), respectively (62). Another phase 3 study combining the AKT inhibitor ipatasertib with abiraterone acetate, which has shown a significant positive impact on PFS in mCRPC patients with PTEN loss, in the 521 (47%) patients who had tumors with PTEN loss (261 in the placebo-abiraterone group and 260 in the ipatasertib-abiraterone group), median rPFS was 16.5 months (95% CI 13.9-17.0) in the placebo-abiraterone group and 18.5 months (16.3-22.1) in the ipatasertib-abiraterone group (hazard ratio [HR] 0.77 [95% CI 0.61-0.98]; p=0.034) (63). Combined AKT and androgen-receptor signaling pathway inhibition is a potential treatment for men with PTEN-loss mCRPC, a population with a poor prognosis. This result suggests that combination therapy targeting multiple genes or pathways may become a major direction for future prostate cancer treatment. Moreover, several natural bioactive compounds including afrocyclamin A, apigenin, arctigenin, curcumin, cryptotanshinone, oridonin, salidroside, and vitexin were reported to target the PI3K/AKT/mTOR pathway, however, some compounds are currently under examination in clinical trials (64).

## 5 WNT

### 5.1 Mechanisms of WNT in cell proliferation and apoptosis

WNT codes a family of proteins involved in the cell signaling process. The WNT signaling pathway is a highly conserved signaling pathway with multiple downstream channels stimulated by the binding of WNT ligand proteins to membrane protein receptors. This pathway plays a crucial in embryonic development, cell proliferation, cell migration and apoptosis. Abnormalities in the WNT signaling pathway are closely associated with the development and progression of various diseases, including cancer (65).

The WNT/β-catenin pathway is the canonical pathway of WNT signaling. Extracellular WNT signaling molecules prevent the phosphorylation of β-catenin, allowing it to accumulate in the cytoplasm. When the concentration of β-catenin in the cytoplasm reaches a certain level, it translocated to the nucleus and combines with the intracellular transcription factor T-cell factor/lymphoid enhancer factor (TCF/LEF) to form a complex. This complex activates the proto-oncogenes Cyclin D1 and c-MYC, leading to tumor cell proliferation, differentiation and maturation (66).

### 5.2 Role of WNT in prostate cancer

Bisson, I. and D.M. Prowse have shown that the WNT/β-catenin signaling pathway is highly active in tumor stem cells and may play a role in the self-renewal of prostate cancer stem cells (67). Wang, B.E., et al. have found that targeting prostate cancer stem cells with WNT/β-catenin signaling inhibitors has been shown to enhance the therapeutic effect of prostate cancer treatments (68). Similar to other genes, WNT signaling is strongly associated with advanced prostate cancer, and Wang, Y., et al. have found that WNT signaling promotes bone metastasis of prostate cancer (69). In addition, β-catenin can interact with other pathways (AR) to coordinate proliferation during tumor growth (70). The above findings suggest that the WNT/β-catenin signaling pathway plays an important role in prostate cancer, especially advanced prostate cancer. This feature may provide a key therapeutic target for the treatment of advanced prostate cancer.

### 5.3 Emerging therapies and challenges

Currently, there is still no effective drug therapy targeting the WNT/β-catenin signaling pathway. However, there are a number of drugs in clinical trials. A new β-catenin mimic small molecule inhibitor, CWP232291, is currently in clinical trials. CWP232291 induce endoplasmic reticulum stress and cell apoptosis, ultimately leading to β-catenin degradation (71). In addition, Cirmtuzumab and Foxy-5 are in Phase 1 trials. Cirmtuzumab is a monoclonal antibody that targets the receptor called ROR1 of the non-canonical Wnt pathway and is suspected to contribute to prostate cancer growth and progression (72). Foxy-5 mimic the effects of Wnt-5a to impair migration of epithelial cancer cells and thereby acting anti-metastatic (73). Given the correlative role of the wnt pathway with AR and MYC, combination therapy with an AR inhibitor or a MYC inhibitor may be useful in the treatment of advanced prostate cancer in the future.

## 6 p53

### 6.1 Mechanisms of p53 in cell cycle and DNA repair

The p53 gene is an important tumor suppressor gene in human cancer, first identified in extracts of transformed cells (74). It plays a vital role in regulating cell cycle and DNA repair. p53 regulate both the G1-S phase (75) and the G2-M arrest (76), thus providing a checkpoint function and repair of genes in the cell cycle. In terms of apoptosis, p53 can induce apoptosis by directly activating its downstream apoptotic genes, such as Bax, Puma and Noxa, etc. in cells that fail to repair DNA damage (77).

### 6.2 Role of p53 in prostate cancer

Deletion of p53 or loss of function due to p53 mutations is detectable in many cancers (78). There are various types of p53 mutations in prostate cancer, including deep deletion, Fusion, shallow deletion, missense mutation, truncating mutation, splice mutation, in-frame mutation and amplification (79). Cotter et al. found that in localized prostate cancer the mutation types of p53 were mainly deep deletion and mutation, while in advanced prostate cancer the mutation types of p53 were deep deletion, mutation and amplification (80). The incidence of p53 mutations is not the same at different stages of prostate cancer, ranging from 31.4% in CRPC to 66.7% in neuroendocrine prostate cancer (81–83). Wang, Y., et al. found that p53 deletion promotes invasion and metastasis in advanced prostate cancer, via enhancing the FAK-Src signaling pathway (84). Actually, p53 mutations occur not only in the advanced stage of prostate cancer but also in its early stage (85). The frequency of these mutations gradually increases as the cancer progresses, reaching the highest level in CRPC (77, 86). These findings suggest that p53 plays a key role in multiple stages of prostate cancer development. In addition, Fonseca, G.N., et al. have shown that the expression of mutant p53 is positively correlated with tumor staging (87). More p53 mutations are found in metastatic prostate cancers than in early-stage prostate cancers, making p53 a potential independent predictor of recurrence of low- and intermediate-grade prostate cancers (88).

In 2006, a study specifically knocked out the Rb and p53 genes in mouse prostate epithelium, and found that after knocking out the Rb gene or p53 gene alone, mice could only develop prostate intraepithelial neoplasia (PIN), but could not develop prostate cancer (89). Only after the simultaneous knockout of Rb and p53 genes, the mice can develop prostate cancer and become highly metastatic (89). It suggests that the loss of Rb and p53 may play a synergistic role in the development and progression of prostate cancer. In a recent study, it was found that in prostate cancer with p53 and Rb deletion, overexpression of the transcription factor SOX2 can mediate lineage plasticity, thereby enabling prostate cancer to evade targeted therapies and lead to CRPC (90). In addition, the deletion of PTEN and p53 can also lead to changes in the lineage of prostate cancer, resulting in the formation of CRPC (91, 92). Ku, S.Y., et al. have found that Rb loss facilitates lineage plasticity and metastasis of prostate adenocarcinoma initiated by PTEN mutation, additional loss of p53 causes resistance to antiandrogen therapy (93). These results indicate that the lineage change of prostate cancer is involved in the deletion of multiple genes, and the specific mechanism of the lineage change of prostate cancer remains to be further studied. This also makes the treatment of advanced prostate cancer more difficult and complex.

Androgen castration is a common treatment for prostate cancer, but most cancers eventually develop androgen independence. Relevant studies have proved that the loss of p53 is associated with CRPC. Inhibition of p53 expression can reduce AR-mediated signal transduction, while overexpression of wild-type p53 can reduce androgen function (94). This is because p53 overexpression inhibits androgen-induced transactivation of NKX3.1 by repressing the promoter of the AR gene and blocking AR-DNA binding activity (95). Therefore, the basic physiological level of wild-type p53 is necessary for AR signal and has a protective effect on it, but the balance between p53 and AR is eliminated as cancer progresses (94), and deletion of p53 leads to androgen-induced transactivation of NKX3.1, which promotes prostate cancer progression. AR also promotes the inactivation of p53. A Study in 2017 showed that AR can induce the translocation of p53 from the nucleus to the cytoplasm via the downstream target gene G3BP2, thereby inhibiting the function of p53 (96).

### 6.3 Emerging therapies and challenges

p53 inactivation may limit the effectiveness of radiation therapy in localized prostate cancer because the effectiveness of treatment relies on p53-mediated cell senescence and apoptosis. Consequently, the p53 pathway can be used as a specific target to enhance the radiosensitivity of prostate cancer cells. For example, using potent radiosensitizers for prostate cancer cells that retain the functional allele of p53 can improve the efficacy of radiation therapy (97). For p53-deficient CRPC, flubendazole is a well-known anti-malarial drug and a potential anti-tumor drug that has been shown to induce cell cycle arrest in the G2/M phase, promote cell death *in vitro* by inducing p53 expression, and inhibit the growth of CRPC tumors in xenograft models (98). But these drugs have had limited clinical trials and their safety has not been proven, there are still many challenges in the treatment of advanced prostate cancer. The findings that p53 interacts with Rb, PTEN and AR in advanced prostate cancer, and synergizes with Rb in the development of prostate cancer, have important implications for the treatment of advanced prostate cancer, and that exploring gene interactions and combining therapies may be of immense help in addressing drug resistance in advanced prostate cancer.

## 7 MYC

### 7.1 Mechanisms of MYC in cell proliferation and apoptosis

The MYC family of proto-oncogenes consists of three homologs: c-MYC (MYC), n-MYC (MYCN), and l-MYC (MYCL), located on chromosomes 8, 2, and 1, respectively. Although MYC family genes encode proteins with similar structural architecture and function, different timing of expression and tissue specificity is exhibited during development (99–101). These genes are involved in regulating integral gene expression and key cellular processes including proliferation, differentiation, cell cycle, metabolism and apoptosis.

### 7.2 Role of MYC in prostate cancer

c-MYC (MYC) is a major promoter of prostate cancer tumorigenesis and progression (102, 103). Under normal conditions, its expression and function are strictly controlled, but overexpression of MYC is frequently observed in prostate cancer (104). Amplification of MYC has been reported to be associated with aggressiveness and poor prognosis in prostate cancer (103). Studies have shown that MYC overexpression in normal luminal cells of the mouse prostate is sufficient to cause PIN and prostate cancer (105, 106). This indicates that dysregulated MYC protein expression is a key oncogenic event driving prostate carcinogenesis. Furthermore, overexpression of MYCN mediates the transformation of CRPC to neuroendocrine prostate cancer (107).

The interplay of MYC with other signaling pathways also exerts a significant role in the development of prostate cancer. Overexpression of MYC leads to the pausing of RNA polymerase II at the promoter-proximal regions of AR-dependent genes, disrupting the AR transcriptional program promote the initiation and progression of prostate tumors (102). Arriaga et al. have recently reported a MYC and RAS co-activation signature associated with metastatic progression and failure to anti-androgen treatments (108). Gretchen et al. found that MYC activation and PTEN deletion in mouse prostate luminal cells induced genomic instability and aggressive prostate cancer in the absence of induced telomere dysfunction or p53 loss of function (109). These studies indicate that MYC can cooperate with other pathways to promote the development of prostate cancer.

### 7.3 Emerging therapies and challenges

Given its key role in prostate cancer, MYC is considered a potential therapeutic target. MYC inhibitors that disrupt MYC and Max dimerization sensitize enzalutamide-resistant prostate cancer cells to growth inhibition by enzalutamide (110). Bromodomain extra-terminal enhancer inhibitors can affect MYC transcription by targeting upstream MYC pathways and have shown preclinical efficacy in MYC-driven CRPC models (111, 112). Kirchner et al. reported that inhibition of PIM, a family of serine-threonine kinase, with the pan-PIM kinases inhibitor AZD-1208 was effective in limiting MYC-driven lesion progression (113). Additionally, a study found that dual inhibitors targeting MYCN and Aurora A kinase (AURKA) could be potential therapies for neuroendocrine prostate cancer (114). Despite these advances, there are still no clinically approved drugs targeting MYC for the treatment of prostate cancer.

## 8 Discussion

In recent years, the incidence of prostate cancer has been steadily increasing. The continuous proliferation and metastasis of prostate cancer cells are critical clinical features and the main causes of mortality in advanced prostate cancer. These processes are regulated by a series of genetic alterations (**Table 1**). It is challenging to elucidate the mechanisms underlying prostate cancer through a single gene mutation or deletion.

The development of prostate cancer involves complex interactions among multiple genes and pathways (**Figure 2**). The molecular mechanisms involving the interaction among multiple genes and pathways remain to be further explored. Further investigation into the synergistic effects of Rb and p53, MYC and PTEN, and WNT and AR in prostate cancer, as well as the identification of common downstream target genes among these interacting genes or pathways, could lead to the discovery of novel targeted therapies. Such research may offer new avenues for treating CRPC and addressing the lineage plasticity of prostate cancer. Currently, resistance to prostate cancer treatment remains a significant challenge. There are many ongoing clinical trials targeting different genes and pathways for the treatment of different stages of prostate cancer, but they still have different limitations, which further suggests that it is critical to explore the interactions of multiple genes and pathways (**Table 2**).

**Table 2:** The clinical trials that are ongoing to treat prostate cancer at different stages.

Gene	PCa (Phase)	CPRC (Phase)	mCRPC (Phase)	Limitations
AR	Apalutamide (2) NCT01790126 Goserelin (2) NCT00298155 ARN-509 (2) NCT01790126 SHR3680 (3) NCT03520478	Enzalutamide (3) NCT00974311 Apalutamide (4) NCT04108208	Enzalutamide (4) NCT02116582 ARV-110 (1/2) NCT03888612 Apalutamide (1) NCT03523442 JNJ-56021927 (1) NCT02162836 TRC253 (1/2) NCT02987829 ARV-110 (1) NCT05177042	Inevitability of castration resistance
Rb (CDK4/6)	Abemaciclib (1/2) NCT05617885	–	Palbociclib (2) NCT02905318 Ribociclib (1/2) NCT02494921 Abemaciclib (2/3) NCT03706365 TQB3616 (2) NCT05156450	Limited clinical trials Questionable safety profile
PTEN (PI3K/AKT)	AZD2014 (1) NCT02064608	LY3023414 (2) NCT02407054 AZD8186 (1) NCT01884285	Perifosine (2) NCT00060437 GSK2636771(1) NCT02215096 Afuresertib (1/2) NCT04060394 Ipatasertib (3) NCT03072238	Limited clinical trials Biomarkers needed for patient selection
WNT	FOXY-5 (1) NCT02020291	–	Cirmtuzumab (1) NCT05156905	Bone-related toxicity Limited clinical trials
p53	PC14586 (1/2) NCT04585750	–	APR-246 (1) NCT00900614 Arsenic trioxide (2) NCT00004149	Limited clinical trials Questionable safety profile
MYC	–	–	ZEN-3694 (2) NCT04471974	Limited clinical trials

The co-deletion of Rb, PTEN and p53 has been shown to confer resistance to antiandrogen therapy. By exploring the molecular mechanisms associated with this co-deletion, we may uncover more effective and sensitive tumor markers and therapeutic targets, thereby improving treatment strategies for advanced prostate cancer. There is still no effective solution to the problem of chemotherapy drug resistance in advanced prostate cancer, but in breast cancer it has been found that drug resistance can be solved through multigene interactions. In HR+/HER2-advanced breast cancer, the medical community has been exploring new therapeutic options for patients who develop resistance after CDK4/6 inhibitors combined with endocrine therapy. Some researchers have found that PI3K pathway inhibitors can alleviate resistance to chemotherapy drug, CDK4/6 inhibitors, in advanced-stage patients. In patients with HR/HER2-advanced breast cancer after progression on the CDK4/6 inhibitor, the patients who applied endocrine therapy in combination with the mTOR inhibitor had a median PFS benefit of 5.1 months (115). This evidence suggests that exploring the mechanisms of multigene interactions could help address chemotherapy resistance in advanced tumors.

In addition to the genes discussed in above, there are a number of genes associated with prostate progression. For example, breast cancer susceptibility gene 1 (BRCA1) and breast cancer susceptibility gene 2 (BRCA2) have been shown to be closely associated with prostate cancer aggressiveness and patient prognosis (116). Both are oncogenes, which can regulate the cell cycle through synergistic effects with other repair mechanisms in the organism and other oncogenes, ensuring the proliferation and apoptosis of normal cells (117). The correlation between BRCA mutation and prostate cancer is still in the research stage, and it is controversial whether BRCA mutation carriers are the high-risk group for prostate cancer, and at present, there is no evidence to show the most suitable method for the treatment of BRCA mutation-associated prostate cancer. Studies have shown that BRCA mutation carriers in the mCRPC population have better treatment outcomes compared to non-carriers, and that patients with either BRCA1 or BRCA2 mutations benefit from treatment with abiraterone or enzalutamide (118). Therefore, exploring the interrelationships of BRCA1 or BRCA2 with other genes and pathways may offer further assistance in the treatment of BRCA mutation-associated prostate cancer.

Src/Ras/extracellular signal-regulated kinase (Erk) pathway also associated with prostate cancer progression. Src is a non-receptor protein tyrosine kinase (119). Src could activate multiple downstream signaling pathways, including the PI3K/AKT pathway and the Ras/Erk pathway, which are important for cell proliferation and DNA synthesis (120, 121). In prostate cancer cells, androgens trigger the binding of AR to Src, this interaction activates Src/Ras/Erk pathway and affects G1 to S cell cycle progression (122). Migliaccio et al. identified an amino acid peptide that inhibits the AR/Src interaction, which inhibits the binding of AR to Src and the activation of the Src/Ras/Erk pathway (123). However, the peptide had no such inhibitory effect in AR-negative prostate cancer cell lines, suggesting that the peptide can only inhibit the androgen receptor-dependent Src pathway in prostate cancer. In addition, Src/Ras/Erk plays an important role in breast cancer, which has led to several studies of Src inhibitors (124). In an ongoing phase 2 trial in prostate cancer, the effect of combining an Src inhibitor with an AR inhibitor versus an AR inhibitor alone on the development of EMT in prostate cancer was compared, but no definitive results have been published (125).

In recent years, the development of immune checkpoint inhibitors (ICIs) has transformed the treatment landscape for various genitourinary malignancies. ICIs are innovative tumor therapeutic agents that restore the body’s anti-tumor immunity by blocking the tumor immune escape mechanism. However, the efficacy of ICIs in prostate cancer remains limited, especially in cases of CRPC, which is challenging to control with traditional therapies. Prostate cancer is often considered an “immune-cold” tumor, characterized by a tumor microenvironment with low immune activity, low tumor mutational burden, interferon signaling dysregulation, and a complex microenvironment, making it less responsive to monotherapy with immunotherapy (126, 127). Recent studies have reported interactions between genetic mutations and immune checkpoints in prostate cancer, indicating that the loss of PTEN and p53 induces the expression of B7-H3, an immune checkpoint molecule, and that elevated B7-H3 contributes to tumor growth and immune suppression of T cells and NK cells in PTEN/p53-deficient tumors (128). Additionally, anti-angiogenesis therapy not only prunes blood vessels essential for cancer growth and metastasis but also reprograms the tumor immune microenvironment (129). Consequently, combination therapy with ICIs and anti-angiogenesis agents can effectively induce tumor regression in some cancer patients. Nevertheless, achieving durable remission remains challenging for advanced prostate cancer patients. Further research has revealed a connection between gene mutations and anti-angiogenic therapy. In prostate cancer, restoring PTEN activity by inhibiting the PI3K-Akt pathway can re-sensitize cancer cells to anti-angiogenic therapy (130). AR can upregulate epidermal growth factor receptor expression in prostate cancer cells (131). These findings suggest that further exploration into the relationship between genomic mutations, immune checkpoints, and anti-angiogenesis may offer innovative approaches to prostate cancer treatment.

The treatment for patients with metastatic prostate cancer includes radiopharmaceuticals in addition to the drugs listed above. There are many types of radiopharmaceuticals used in mCRPC patients. Strontium-89 (89Sr) has been shown to be very effective in the treatment of patients with chemotherapy-refractory bone metastases (132). Samarium-153 (153Sm) lexidronam (EDTMP) has also been shown to provide significant pain relief in patients with bone metastases (133). The most recent radiopharmaceutical available is lutetium-177 (177Lu). The newest radiopharmaceutical currently on the market is lutetium-177 (177Lu)-PSMA-617, which was approved on 23 March 2022 by the US Food and Drug Administration. Patients are eligible for this treatment if they have mCRPC, have been previously treated with Androgen pathway inhibitors (ARPI) and type chemotherapy, and have positive prostate-specific membrane antigen (PSMA) imaging, indicating PSMA expression in metastatic lesions (134). More research into PSMA-targeted therapies is currently underway. Over the next decade, radiopharmaceuticals may play a central role in the treatment of patients with advanced prostate cancer.

## 9 Conclusion

Rb, PTEN, WNT, p53, MYC and AR and their interactions play important roles in regulating prostate cancer development. Investigating the mechanisms of interaction between various pathways and genes can help to identify new common target genes and provide more effective therapeutic strategies to address drug resistance in CRPC. In addition, treatment of these genes and pathways in combination with immune checkpoints, anti-angiogenesis and radiopharmaceuticals may offer innovative approaches to prostate cancer treatment. Such insights could inform the selection of therapeutic strategies, thereby establishing a robust foundation for the treatment of prostate cancer.
